# Exploring the Multifaceted Role of Scientific Paper and Poster Presentations: A Narrative Review

**DOI:** 10.7759/cureus.96314

**Published:** 2025-11-07

**Authors:** Sayem A Mulla, Amit Patil, Sheetal Mali, Himmat Jaiswal, Hitesh R Sawant, Pooja Shah, Vyshnavi Mundada

**Affiliations:** 1 Dentistry, Bharati Vidyapeeth (Deemed to be University) Dental College and Hospital, Navi Mumbai, IND; 2 Conservative Dentistry and Endodontics, Bharati Vidyapeeth (Deemed to be University) Dental College and Hospital, Navi Mumbai, IND; 3 Orthodontics and Dentofacial Orthopedics, Bharati Vidyapeeth (Deemed to be University) Dental College and Hospital, Navi Mumbai, IND

**Keywords:** academic communication, continued education, higher education, poster presentation, research dissemination, scientific writing, skill development

## Abstract

In order to improve education, foster professional growth, and facilitate the dissemination of research, scientific publications and poster presentations are crucial elements of academic communication. Their diverse responsibilities are particularly important given how higher education and interdisciplinary research are developing. A qualitative synthesis of the literature gathered from databases such as PubMed, Google Scholar, and Scopus was used to perform this narrative review. To glean thematic insights on the functions and advantages of scientific writing and presentations, pertinent English-language publications over the last 20 years were examined, including guidelines, academic commentary, and relevant articles. Posters and scientific publications are powerful resources for academic communication, professional involvement, and in-depth study. They facilitate the dissemination and critique of research findings, encourage teamwork through multi-author contributions, support ongoing education through literature exploration and content synthesis, develop critical, creative, and technical skills, and play a crucial role in postgraduate and doctoral education. Engaging in these kinds of activities has also been connected to improved academic achievement and professional growth. Across fields, scientific articles and poster presentations are essential and interrelated to people's academic and professional development. To develop a capable, research-focused, and internationally competitive academic community, their inclusion and promotion in academic curriculum and institutional culture are essential. The aim of this paper is to investigate and clarify the many roles and effects of scientific publications and poster presentations in the fields of research, teamwork, skill development, advanced academic training, and continuing education.

## Introduction and background

In fields that rely heavily on research, scientific papers and poster presentations remain central to the dissemination of research findings in academia. The mainstays for sharing discoveries, promoting intellectual conversation, and spreading information among the many forms of academic communication are scientific publications and poster presentations [1]. These presentation formats reflect an ongoing cycle of investigation, analysis, synthesis, and distribution rather than just the display of data or the act of publication. The use of these communication techniques has grown throughout time to encompass not just the presentation of new information but also the critical evaluation of previously held beliefs, theoretical developments, and even early research [2].

**Figure 1:**
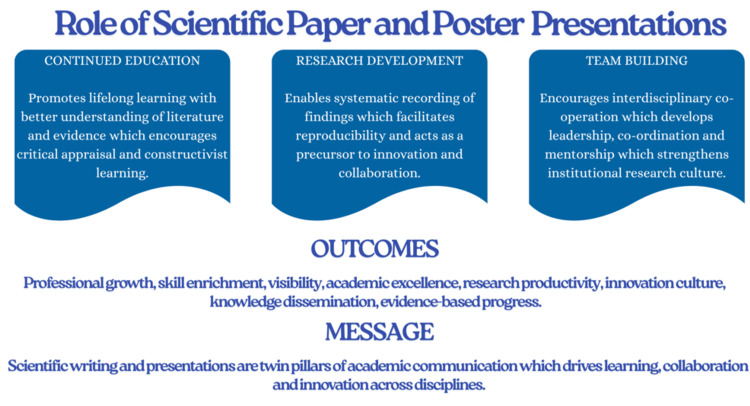
Multifaceted role of scientific paper and poster presentations. Image Credit: Sayem A. Mulla, Author

The functions of scientific publications and posters have changed as research grows more collaborative and multidisciplinary [3]. They are essential in recording evidence-based findings, but they also help to advance professional skills, foster cooperation, support lifelong learning, and raise the bar for higher education [4]. This narrative study explores each of these aspects in depth, highlighting the importance of writing and presenting scientific work for the advancement of people and organizations in the academic and clinical domains (Figure 1).

What are scientific papers and posters?

Research findings, literature reviews, or discussions of novel theories or methods are the main goals of scientific papers, which are organized publications. Peer-reviewed journals, which serve as filters to guarantee scientific rigor and relevance, publish these publications [5]. The purpose of the Abstract, Introduction, Methodology, Results, Discussion, and Conclusion sections of a typical scientific publication is to provide information in a logical and repeatable manner. Original research articles, systematic or narrative reviews, meta-analyses, case reports, letters to the editor, and technical briefs are among the several forms of scientific writings that are recognized by the academic world [6].

Simultaneously, poster presentations function as a visual communication tool in which research is presented in a compact manner through the use of graphs, charts, photographs, and brief text summaries. Posters are frequently utilized in scientific and academic conferences, where they are displayed during special sessions that facilitate audience-presenter interaction [7]. The Council of Science Editors states that early-stage researchers benefit greatly from poster presentations as they offer a less formal framework for academic discussion and direct feedback than oral presentations [8].

Together, scientific publications and posters are how researchers present their findings to the general public. This includes sharing scientific justification, methodology, theoretical ideas, and wider ramifications in addition to results presentation [9]. The same study assumes an interactive dimension when presented verbally or graphically, promoting inquiries, criticism, and more idea development. Under these circumstances, scientific communication turns into an iterative and cyclical process that is ingrained in academic culture [10].

## Review

Methodology

An in-depth literature search was carried out on scholarly databases such as PubMed, Scopus, and Google Scholar. The keywords used included "scientific papers" and "scientific posters" in various combinations of Boolean operators. Studies and articles published in the previous 20 years and available in the English language were included. The initial search results were then screened by three reviewers (SAM, AP, and SM) for their relevance. After careful assessment, a total of 55 articles were included in this narrative synthesis.

Role in continued education

The process of learning new things during one's career is known as continuing education. The necessity for ongoing education is particularly pressing in scientific fields because of the rapid advancements in technology, clinical procedures, and research [11]. Because they need in-depth knowledge of current literature, research techniques, and analytical tools, scientific publications and poster presentations are crucial to this process [12].

Finding a research topic of interest is the first step in creating a scientific paper or poster. This is followed by thorough literature searches, frequently conducted using databases like PubMed, Scopus, or Embase [13]. The learner's comprehension of current trends, gaps in the literature, and methodological improvements is widened only by this. For instance, by studying related cases and pertinent recommendations, a medical student working on a case report gains knowledge about the ailment being reported as well as its epidemiology, clinical presentation, diagnostic procedures, and treatment methods [14].

Further knowledge of the subject is also required in order to present the paper or poster at a seminar or conference. The presenter needs to be ready to answer queries, explain findings, and defend the approach. Constructivist theories of learning, which contend that knowledge is best assimilated when students actively participate in its development and dissemination, are consistent with this active involvement [15].

When compared to passive learning modalities, research by Cantillon and Wood (2010) highlights that self-directed learning, like creating a scientific presentation, leads to greater retention rates and deeper understanding [16]. As a result, scientific writing and presentation greatly aid in professional growth and guarantee that experts continue to be lifelong learners who can adjust to the rapidly evolving fields of research and medicine [17].

Role in research

Research is the methodical study done to find and understand new information. Every stage of the research process, from formulating hypotheses to drawing conclusions, is recorded in writing and presented in the scientific sphere [18]. Scientific publications contribute to the accumulation of knowledge by offering a structured means of archiving research findings for present and future use [19].

Ensuring reproducibility is one of a scientific paper's main purposes. Other researchers can verify, duplicate, or expand on previous work, thanks to the thorough description of procedures and findings. Precise and open reporting offered by scientific writing is essential to the iterative character of science [20]. Additionally, articles published in indexed journals like those found in Medline, Web of Science, or Scopus are read by people all over the world and frequently referenced, which boosts the researcher's academic reputation and institutional metrics [21].

In the lifecycle of scientific communication, oral presentations and posters are equally important [22]. They act as venues for the first distribution of discoveries, frequently prior to formal peer-reviewed publication, even if they do not necessarily provide indexed articles [23]. This makes it possible for researchers to get input, find possible partners, and look into financing possibilities. Results at a scientific meeting, for example, can be improved upon in response to criticism and then submitted as a paper to a prestigious journal [24].

Papers presented at international conferences have a better chance of being approved for journal publication and tend to earn more citations. This demonstrates how written and visual/oral scientific communication are interdependent in the dissemination of knowledge and innovation [25].

Role in team building

Rarely are scientific investigations and their dissemination lonely pursuits. The procedure usually involves several participants with different responsibilities and levels of skill, from data gathering to article preparation and poster design [26]. In interdisciplinary research, when each team member contributes a distinct perspective, clinical, statistical, methodological, or conceptual, collaborative authorship has become the standard [27].

Working as a team to co-author a paper or create a poster promotes the growth of professional and interpersonal abilities, including shared accountability, coordination, delegating, and dispute resolution. These cooperative exercises foster respect and understanding between mentors and peers. Additionally, collaborative writing promotes a mentorship culture in academic organizations and allows junior researchers to gain knowledge from more seasoned colleagues [28,29].

A sense of collective ownership and pleasure in the finished product is also fostered by taking part in collaborative poster presentations or multi-authored articles. Because it exposes undergraduate and graduate students to academic obligations and expectations early in their careers, this may be extremely motivating [30]. Group research projects can greatly enhance students' professional attitudes, analytical thinking, and communication abilities [31]. Team-based paper and poster activities frequently result in the creation of research cells or working groups in institutional settings, such as hospitals or universities, so reinforcing the function of scientific communication as a tool for leadership development and team building [32].

Role in skill development

The process of creating and presenting scientific knowledge helps people acquire a wide range of abilities that go beyond the realm of science. These encompass both hard and soft skills, all of which are necessary for success in the classroom and in the workplace [33].

Analytical and Critical Thinking

By methodically examining and integrating the body of scientific knowledge, literature reviews help researchers develop logical thinking. Finding knowledge gaps, assessing the reliability of sources, and making linkages between various research are all steps in this process [34]. Researchers can identify biases, evaluate validity, and improve conceptual understanding by critically comparing methods and results. Their capacity to develop strong arguments based on facts rather than conjecture is strengthened by such intellectual activities. As a result, scientific thinking and evidence-based decision-making are founded on literature reviews [35].

Through the promotion of objective analysis and logical reasoning, data interpretation and hypothesis testing further strengthen critical evaluation abilities. Complex datasets need interpretation, pattern recognition, and the determination of whether observed results confirm or disprove a theory. By using statistical techniques, controlling for variables, and taking potential mistakes into account, this procedure promotes methodological rigor and analytical precision [36]. Regular participation in this cycle of scientific reasoning fosters skepticism and intellectual curiosity, two qualities essential to an autonomous thinker. In the end, these methods help researchers become more capable of assessing their findings in larger scientific settings and making significant contributions to knowledge advancement [37]. *Writing and Documentation*

Maintaining consistency, trustworthiness, and traceability in research communication requires a thorough awareness of academic formatting rules like the American Psychological Association (APA) or Vancouver styles, which are instilled by structuring a scientific document [38]. This procedure ensures openness and ethical integrity by teaching researchers how to correctly reference prior work, credit sources, and structure sections in a methodical manner. Following these guidelines improves the findings' repeatability and gives readers the confidence to critically assess methods and conclusions [39].

Furthermore, a paper's structure encourages accuracy, lucidity, and logical arrangement of concepts, all of which are essential components of scientific writing. It teaches writers how to formulate hypotheses, clearly communicate facts, and analyze findings [40]. The focus on clear communication and logic supported by facts lessens prejudice and enhances readability. In the end, this field improves a researcher's capacity to successfully communicate intricate scientific ideas to a range of audiences, advancing and disseminating trustworthy scientific information [41]. *Communication and Public Speaking*

Discussions over posters and oral presentations are lively forums that improve a researcher's capacity to communicate intricate scientific concepts in an interesting and straightforward manner. To convert complex data into formats that are understandable for a range of audiences, they need to integrate verbal, visual, and analytical talents [42]. Critical thinking, narrative coherence, and the thoughtful application of visual aids to highlight important discoveries are all encouraged by this procedure. Presenters can better make research powerful and clear by reducing technical jargon and emphasizing conceptual clarity [43].

These interactive forms also encourage quick feedback, active knowledge sharing, and the defense of scientific arguments in front of experts and peers. Under scholarly analysis, such involvement enhances self-assurance, spontaneity, and flexibility in communication [44]. Oral sessions improve public speaking and reasoning abilities, whereas poster sessions, in particular, promote succinct yet compelling data presentation. When taken as a whole, these experiences help researchers improve their scientific literacy and rhetorical proficiency, which are essential for promoting successful science communication and career advancement [45].​​​​​​​ *Technical Proficiency*

Digital literacy is the foundation when contemporary scientific research is concerned. It enables the researchers to effectively gather, examine as well and comprehend complicated data. Programs like IBM SPSS Statistics (IBM Corp., Armonk, New York, United States) and GraphPad Prism (Dotmatics, Boston, Massachusetts, United States) improve statistical proficiency by making it possible to handle huge datasets, carry out multivariate analyses, and precisely visualize findings [46]. Due to these technologies, evidence-based decision-making can be done since human errors are reduced.

Furthermore, digital competency encompasses more than just data analysis; it also includes the efficient administration and display of scientific data. LaTeX (The LaTeX Project, Mainz, Germany) makes it possible to create excellent, organized scientific papers with mathematical accuracy, while EndNote (Clarivate Plc, London, United Kingdom) makes it easy to handle citations and references, guaranteeing academic integrity and conformity to publishing standards. When combined, these platforms develop a technologically proficient researcher who can produce rigorous, open, and widely shared scientific work [47].​​​​​​​ *Creativity and Visual Design*

The creation of posters in scientific communication fosters creativity and clarity in the presentation of research findings by fusing creative and analytical abilities. Researchers can emphasize important themes while preserving a cogent narrative flow by using information arrangement principles to present data logically and attractively [48]. By fostering an awareness of hierarchy, balance, and geographical distribution, this approach makes difficult material understandable and interesting. Good content organization also improves audience understanding and retention, which is important for sharing scientific concepts [49].

A researcher's capacity to produce aesthetically pleasing and scientifically correct posters is further enhanced by the application of color theory and design concepts. Proper color schemes help with information remember by influencing perception and emotional reaction in addition to drawing attention [50]. Visual harmony and readability are enhanced by design elements including contrast, alignment, and proportion [51]. When combined, these components turn scientific facts into a convincing, aesthetically pleasing, and educational medium. As a result, making posters turns into a dynamic exercise in scientific innovation and superior communication.

The process of writing and presenting scientific work requires skill development, which is not an afterthought. Undergraduate researchers who participate in scientific presentations can enhance academic performance, develop more confidence in public speaking, and have a stronger desire to pursue research careers [52].

Role in advanced education

Scientific papers and presentations are now required as part of the curriculum and degree requirements in higher education, particularly at the postgraduate and doctoral levels. These assignments serve as benchmarks for academic advancement, demonstrating the student's capacity for autonomous research and communication [53]. The significance of publications and conference presentations in postgraduate education has been underlined by academic institutions and regulatory agencies in India, including the University Grants Commission (UGC), Dental Council of India (DCI), and Medical Council of India (MCI). Furthermore, research output is being employed more and more as a criterion for faculty evaluation and institutional ranking [54].

Additionally, scientific presentations give academics and students the chance to apply for postdoctoral or advanced fellowships, network with specialists, and access new funding options. Institutions make sure that students graduate with the theoretical knowledge, practical skills, and experience required for academic competitiveness on a worldwide scale by incorporating these academic activities into the basic framework of higher education [55].

## Conclusions

Scientific publications and poster presentations are essential to professional growth and scientific progress; they are not only academic etiquette. Every player in the academic ecosystem, from novices to seasoned researchers, gains from writing and presenting scientific findings. Distribution of research, teamwork, skill development, and educational growth are greatly aided by these activities. Therefore, in order to foster a culture of inquiry, excellence, and innovation, institutions must keep encouraging, supporting, and rewarding scholarly communication efforts. 

This review's main takeaway is that scientific articles and poster presentations are dynamic instruments that promote information sharing, skill development, teamwork, and lifetime learning far more than just academic requirements. Together, they serve as the cornerstone of successful scientific communication, connecting the creation of research with learning and career advancement. These platforms support the development of a research-focused academic culture that advances both individual careers and institutional greatness by encouraging critical thinking, collaboration, and creativity.
